# Electronic cigarette and moist snuff product characteristics independently associated with youth tobacco product perceptions

**DOI:** 10.18332/tid/125513

**Published:** 2020-08-28

**Authors:** Benjamin W. Chaffee, Elizabeth T. Couch, Janelle Urata, David Cash, Miranda Werts, Bonnie Halpern-Felsher

**Affiliations:** 1 Division of Oral Epidemiology and Dental Public Health, University of California San Francisco, San Francisco, United States; 2 Division of Adolescent Medicine, Stanford University, Stanford, United States

**Keywords:** adolescent health, electronic cigarettes, smokeless tobacco, risk perceptions, tobacco regulatory science

## Abstract

**INTRODUCTION:**

Tobacco product characteristics convey product attributes to potential users. This study aimed to assess independent contributions of specific e-cigarette and smokeless tobacco product characteristics to adolescents’ perceptions about these products.

**METHODS:**

In 2019–2020, students (N=1003) attending a convenience sample of 7 high schools in California (USA) were individually randomized to one of two discrete choice experiments, featuring either electronic cigarettes (e-cigarettes) or moist snuff. Participants were presented like-product pairs of randomlygenerated hypothetical tobacco products differing in device type, flavor, vapor cloud, and nicotine amount (for e-cigarettes) or differing in brand, flavor, cut, and price (for moist snuff). Within pairs, participants were asked about which product they were more curious, was more dangerous, would give a greater ‘buzz,’ and would be easier to use. Conditional logistic regression was used to quantify independent associations of product characteristics to participants’ choices.

**RESULTS:**

Each e-cigarette and moist snuff characteristic was independently associated with multiple product perceptions. All non-tobacco flavors were associated with more curiosity and perceived ease-of-use but lower perceived danger. Tank and pod-type e-cigarettes were viewed as easier to use and garnered more curiosity than ‘cigalike’ or ‘drip-mod’ devices. Smaller vapor cloud e-cigarettes and lower-price moist snuff were viewed as less dangerous, less buzz-inducing, and easier to use. Product ever users held stronger perceptions than never users about device type (e-cigarettes) and brands (moist snuff), while product naïve participants more strongly associated flavor with danger and buzz.

**CONCLUSIONS:**

Tobacco product characteristics convey product attributes to adolescents that may increase appeal. Restricting specific characteristics, including flavors, could reduce positive perceptions of these products among youth.

## INTRODUCTION

Use of non-cigarette tobacco is increasing among youth. Past 30-day use of electronic cigarettes (e-cigarettes) among US high school students recently rose substantially, more than doubling in two years, from 11.7% in 2017 to 27.5% in 2019^1,2^. Similarly, the use of conventional smokeless (spit) tobacco in 2018 nearly equaled the prevalence of cigarette smoking among male US high school students (smokeless: 8.4%; cigarettes: 8.8%)^[Bibr cit0001]1^. Use of e-cigarettes and smokeless tobacco exemplify a larger trend, in which a broadening range of non-cigarette and non-combustible tobacco products threatens to erode public health gains in reducing youth tobacco use^1^.

Tobacco product characteristics, such as flavors, nicotine strength, e-cigarette device type (e.g. refillable tank or pod), or smokeless tobacco cut (e.g. long cut or pouched snuff), can signal properties of tobacco products to potential consumers, including youth. Perceived properties might relate to the taste, potency, or relative safety of the product. To the extent that specific tobacco product characteristics lead to youth viewing certain tobacco products as more appealing or associated with fewer risks, those characteristics represent plausible targets of regulation or other restrictions intended to reduce youth use.

A combination of branding, product design, and real or perceived properties likely operate individually and collectively to shape youth tobaccorelated attitudes and decision-making. Research that identifies and quantifies the contributions of specific tobacco product characteristics is potentially appealing to regulators seeking to reduce youth use without outright bans on entire classes of products. Discrete choice methods stem from economic theory that consumer preferences are based on the multiple intrinsic characteristics of goods or products^3^, and have recently been applied to tobacco control and tobacco regulatory science^4^.

Discrete choice experiments are designed to identify the independent contributions of component parts of a good or service to potential consumers’ overall preferences and/or beliefs. In surveys, participants are often asked to choose between two different products or scenarios, each representing a composite set of relevant attributes at varying levels (e.g. price, amount, flavor), allowing quantification of how these characteristics independently contribute to respondents’ choices. Recent work has examined adults’ preferences related to waterpipe tobacco^5^ and e-cigarettes^6^, as well as youth e-cigarette preferences^7^. In the latter study^7^, youth were more likely to prefer e-cigarettes with non-tobacco flavors and less likely to choose products with Food and Drug Administration (FDA) warning labels or ‘cigalike’ (cigarette-like) devices.

The present study expands on previous discrete choice studies by including a community-based sample of youth, assessing both e-cigarettes and smokeless tobacco, and considering multiple specific perceived properties, such as danger and ease of use. The study objective is to evaluate the extent to which specific characteristics of e-cigarette and moist snuff smokeless tobacco products convey product qualities to youth, especially those perceived qualities that may lead to greater youth appeal and product use. Such product characteristics are plausible targets of potential FDA regulation or local policy designed to reduce youth tobacco use. Based on prior work showing favorable perceptions and disproportionately higher use levels of flavored tobacco (non-tobacco flavor such as mint, menthol, fruit or candy) use among youth and young adults^8-10^, we hypothesize that flavored tobacco, independent of other product characteristics, will be associated with greater curiosity and ease-of-use but lower perceived danger and potency, both for e-cigarettes and moist snuff. As an exploratory objective, we additionally examine differences in the association between product attributes and youth perceptions by gender and tobacco use status.

## METHODS

### Study design

This discrete choice experiment was embedded in the UCSF Adolescent Tobacco and Health Study, an in-person, school-based survey of high school students recruited from grades 9 and 10 in Northern and Central California. The overall survey included items about current and past tobacco use, perceptions of new and emerging tobacco products, socio-environmental variables, health conditions, and use of other substances as part of an overarching goal to assess factors influencing tobacco-related behaviors over time in this population. Thus, the present analysis is crosssectional (all data collected at one timepoint) and experimental (responses were recorded to scenarios generated and randomly displayed in the survey). An Institutional Review Board at the University of California San Francisco reviewed and approved all study procedures. Participating students received a $10 gift card to an online retailer. Participating schools received $300.

### Study enrollment

Overall study enrollment and survey administration took place from March 2019 to February 2020 at 8 public high schools. Due to limited classroom time, the final school completed a shortened questionnaire that excluded discrete choice items. Thus, 7 schools recruited from March 2019 to January 2020 were included. Eligible schools were located in municipalities with fewer than 50000 residents and in counties of population density less than 1000 persons/ square-mile^11^. Schools were selected for participation via purposeful sampling that targeted counties with expected higher levels of tobacco use and where the investigative team had existing collaborative research relationships. All grade 9 and 10 students at participating schools were eligible to participate. Study staff visited all sessions of a required course (e.g. World History) to explain study objectives and distribute parental consent and student assent forms, followed by in-class administration of the electronic survey on computers 1–2 weeks later.

### Discrete choice experiments

As a programmed feature of the survey software (Qualtrics, SAP, Provo, UT), students were randomized at the participant level with equal probability to one of two discrete choice experiments: e-cigarettes or moist snuff smokeless tobacco. Participants randomized to the e-cigarette experiment were presented six pairs of randomly generated hypothetical e-cigarette products (in six separate, consecutive items) under a full factorial design. The composite products differed in device type (cigalike, tank, drip-mod, pod), flavor (tobacco, dessert, fruit, mint, ‘unicorn’), vapor cloud (large, small), and nicotine amount (none, low, moderate, high). Prior to viewing the computergenerated composite e-cigarettes, participants were shown an image containing the possible e-cigarette product characteristics they might see (Supplementary file, Figure A1).

Participants randomized to the smokeless tobacco discrete choice experiment were presented six separate, consecutive pairs of randomly generated hypothetical moist snuff products under a full factorial design. The composite products differed in brand (Copenhagen, Grizzly, Longhorn, Skoal), flavor (tobacco, wintergreen, mint, fruit), cut (fine, long, wide, pouch), and price ($3, $5, $8). Prior to viewing the computer-generated composite moist snuff products, participants were shown an image containing the possible moist snuff product characteristics they might see (Supplementary file, Figure A2).

The number of displayed characteristics and their levels were necessarily constrained to avoid excessive cognitive burden. Some characteristics were productspecific (e.g. e-cigarette vapor cloud and moist snuff cut). Prioritizing which characteristics to retain for each product was based on existing qualitative and quantitative literature on youth tobacco-related perceptions and use motivations^7,12,13^.

In each experiment, for each pair of product composites, participants were asked which product ‘are you more curious about’, which ‘is more dangerous to health’, which ‘would be easier to use’, and which ‘would give a bigger 'buzz' or 'head rush'’. These outcomes were chosen because of previous work showing associations between tobacco use and/or susceptibility with youth-reported curiosity^14^, perceived danger^15^, and perceived ease-of-use^16,17^. The outcome ‘buzz’ was introduced to measure perceived physiological effects or potency. Participants could select either composite product within the pair or ‘neither of these options’. Supplementary file, Figure A3 shows an example question layout.

Of 1052 eligible participants, 525 took part in the e-cigarette discrete choice experiment and 522 the moist snuff discrete choice experiment (5 did not complete any discrete choice items). Participants providing ‘straight-line’ responses with no variation in choosing the lefthand-side or righthand-side product (n=44) were excluded to improve data quality, leaving 495 in the e-cigarette experiment and 508 in the moist snuff experiment.

### Statistical analysis

Conditional logistic regression was used to quantify the independent contribution of product attributes (characteristics) to participants’ choices while maintaining the matching of each pair. The position of the composite product on the screen (left or right) was also included in models to account for possible ordering preference. A positive regression coefficient indicates how much the attribute level in question (e.g. flavor: fruit) increased the log-odds of that composite product being chosen relative to the reference level (e.g. flavor: tobacco), holding all other product attributes constant. Negative coefficients indicate how much that characteristic independently decreased the log-odds of being chosen. All models used the cluster-robust variance option (Stata 16.0, StataCorp, College Station, TX) to account for multiple items per participant.

Interaction terms were added to models to assess differences according to participant gender (male or female; not identifying as male or female excluded: <2% of sample) and history of tobacco product use (ever or never, for e-cigarettes or smokeless tobacco, according to the product experiment). Differences by gender and tobacco use were assessed in separate models. Likelihood ratio tests were performed to assess the overall improvement in model fit by adding interaction terms. Both main effects and interactions were considered statistically significant if 95% confidence intervals excluded the null value, without adjustment for multiple hypothesis tests.

## RESULTS

Participants in the e-cigarette discrete choice experiment and in the smokeless tobacco experiment did not differ from each other in their aggregate demographic characteristics or tobacco use (Table 1). Approximately half the sample identified as female, Hispanic/Latinx, and as eligible for free or reducedcost school lunch (Table 1). E-cigarettes were the most commonly used tobacco product (37% ever use), whereas a smaller percentage used smokeless tobacco products (8% ever use).

**Table 1:** Characteristics of the study sample, adolescents in rural California, USA, 2019–2020 (N=1052)

*Characteristics*	*Overall ^a^ (N=1052) %*	*E-cigarette sample (N=495) %*	*Moist snuff sample (N=508) %*	*Excluded ^b^ (N=49) %*	*p ^c^*
**Grade in school**					0.78
Ninth	56.9	58.1	55.8	56.5	
Tenth	43.1	41.9	44.2	43.5	
**Gender**					0.90
Female	53.4	53.5	53.4	51.0	
Male	45.2	44.8	45.5	46.9	
Other/decline	1.3	1.6	1.0	2.0	
**Race/ethnicity**					0.002^d^
Hispanic/Latinx	53.1	51.1	56.3	40.8	
Non-Hispanic White	34.1	36.2	32.7	28.6	
Other/unknown	12.7	12.7	11.0	30.6	
**National School Lunch Program**					0.88
Free/reduced-price	54.2	52.9	55.4	55.3	
Full price	31.7	32.2	31.0	34.0	
Don't know	14.1	14.9	13.5	10.6	
**Substance ever use**					
E-cigarettes	36.9	38.0	36.0	34.7	0.77
Smokeless tobacco^e^	7.9	7.1	8.7	8.2	0.64
Any tobacco^f^	41.2	41.8	41.0	36.7	0.78
Cannabis	33.6	33.7	33.9	28.6	0.75
**Substance past 30-day use**					
E-cigarettes	18.1	18.4	18.5	10.2	0.34
Smokeless tobacco^e^	1.9	1.8	1.8	4.1	0.52
Any tobacco^f^	20.0	19.8	20.7	14.3	0.56
Cannabis	18.1	17.2	19.1	16.3	0.69

a Includes all participants at the 7 schools where discrete choice questions were posed. Number of observations may be less than the total for some variables due to missing data.

b Did not complete any discrete choice items (n=5) or provided same response on every item (‘straight-line’ pattern).

c Chi-squared test for global difference over three groups (e-cigarette sample, moist snuff sample, excluded).

d Pairwise chi-squared tests comparing e-cigarette sample versus moist snuff sample (p=0.25).

e Includes moist snuff, chewing tobacco, or snus.

f Include e-cigarettes, smokeless tobacco, cigarettes, cigars, or hookah.

In the e-cigarette experiment (Table 2), tank-type and pod-type devices garnered more curiosity and were perceived as easier to use than cigalike or dripmod devices. Relative to tobacco flavor, all flavors were associated with more curiosity, less perceived danger, and greater perceived ease-of-use. On the adjusted log-odds scale, where tobacco flavor is the reference, fruit (coefficient: 1.04; 95% CI: 0.78, 1.30) and dessert (0.92; 95% CI: 0.66, 1.19) were most positively associated with curiosity, while mint (-0.32; 95% CI: -0.49, -0.14) and unicorn (-0.28; 95% CI: -0.45, -0.10) were the flavor options most negatively associated with danger. Smaller vapor cloud e-cigarettes were viewed as less dangerous, offering less buzz, and easier to use. Nicotine amount was strongly associated with perceived danger and buzz. High nicotine devices were viewed with less curiosity, as more dangerous, delivering more buzz, and less easy to use, relative to low nicotine or nicotine-free devices (Table 2).

**Table 2:** Perceptions associated with e-cigarette product characteristics, a discrete choice experiment among adolescents in rural California, USA, 2019–2020 (N=495)

	*Curiosity Coefficient (95% CI)*	*Danger Coefficient (95% CI)*	*Buzz Coefficient (95% CI)*	*Ease of use Coefficient (95% CI)*
**Device type**				
Cigalike	Ref.	Ref.	Ref.	Ref.
Tank	0.40 ( 0.19, 0.61)	-0.07 (-0.23, 0.08)	0.16 ( 0.00, 0.32)	0.23 ( 0.05, 0.41)
Drip-mod	0.06 (-0.16, 0.27)	0.05 (-0.11, 0.20)	0.16 ( 0.00, 0.33)	-0.14 (-0.32, 0.04)
Pod	0.37 ( 0.17, 0.57)	0.01 (-0.15, 0.17)	0.07 (-0.09, 0.23)	0.55 ( 0.39, 0.72)
**Flavor**				
Tobacco	Ref.	Ref.	Ref.	Ref.
Dessert	0.92 ( 0.66, 1.19)	-0.24 (-0.42, -0.06)	-0.08 (-0.26, 0.10)	0.40 ( 0.21, 0.60)
Fruit	1.04 ( 0.78, 1.30)	-0.25 (-0.44, -0.07)	-0.11 (-0.29, 0.08)	0.48 ( 0.29, 0.67)
Mint	0.77 ( 0.50, 1.04)	-0.32 (-0.49, -0.14)	-0.15 (-0.33, 0.03)	0.35 ( 0.16, 0.54)
Unicorn	0.61 ( 0.35, 0.88)	-0.28 (-0.45, -0.10)	-0.13 (-0.31, 0.04)	0.27 ( 0.08, 0.46)
**Vapor cloud**				
Large	Ref.	Ref.	Ref.	Ref.
Small	-0.04 (-0.18, 0.10)	-0.31 (-0.43, -0.20)	-0.40 (-0.52, -0.28)	0.27 ( 0.15, 0.39)
**Nicotine amount**				
None	Ref.	Ref.	Ref.	Ref.
Low	-0.17 (-0.35, 0.01)	0.85 ( 0.65, 1.04)	0.58 ( 0.39, 0.78)	-0.16 (-0.32, 0.00)
Moderate	-0.32 (-0.52, -0.12)	1.59 ( 1.39, 1.79)	1.18 ( 0.97, 1.38)	-0.41 (-0.59, -0.23)
High	-0.51 (-0.74, -0.29)	2.15 ( 1.92, 2.38)	1.75 ( 1.52, 1.98)	-0.54 (-0.73, -0.35)
**Position**				
Left	Ref.	Ref.	Ref.	Ref.
Right	-0.04 (-0.17, 0.09)	0.04 (-0.06, 0.13)	0.04 (-0.05, 0.13)	0.06 (-0.04, 0.15)
				

Table reports coefficients from conditional logistic regression models in a discrete choice experiment. Coefficients indicate how much the attribute level in question (e.g. flavor: fruit) increased or decreased the log-odds of a displayed e-cigarette product being chosen relative to the reference level (e.g. flavor: tobacco), adjusted for all other displayed e-cigarette attributes. For each displayed pair of e-cigarettes, participants were asked about which they were more curious, and which was more dangerous, would give a greater buzz, and would be easier to use. Positive values indicate characteristics (relative to reference) that independently contributed to greater curiosity or perceived danger, buzz, and ease of use, respectively. CI: confidence interval. Ref.: reference.

In the moist snuff experiment (Table 3), one brand was perceived as the most dangerous but also the easiest to use. Relative to tobacco flavor, all moist snuff flavors were associated with more curiosity, less perceived danger, and greater perceived ease-ofuse. On the adjusted log-odds scale, fruit (1.18; 95% CI: 0.86, 1.51) and mint (1.10; 95% CI: 0.77, 1.42) flavors were the characteristic levels associated with the most curiosity, while fruit flavor was also viewed as offering the least buzz (-0.33; 95% CI: -0.51, -0.15). Associations of modest magnitude suggested that finecut products were perceived as less dangerous and offering less buzz. Higher price products were viewed with more curiosity, as more dangerous, offering a greater buzz, and being less easy to use (Table 3).

**Table 3:** Perceptions associated with moist snuff smokeless tobacco product characteristics, a discrete choice experiment among adolescents in rural California, USA, 2019–2020 (N=508)

	*Curiosity Coefficient (95% CI)*	*Danger Coefficient (95% CI)*	*Buzz Coefficient (95% CI)*	*Ease of use Coefficient (95% CI)*
**Brand**				
Copenhagen	Ref.	Ref.	Ref.	Ref.
Grizzly	0.03 (-0.22, 0.28)	-0.28 (-0.45, -0.11)	-0.12 (-0.28, 0.04)	-0.30 (-0.47, -0.14)
Longhorn	-0.12 (-0.39, 0.14)	-0.21 (-0.37, -0.06)	-0.14 (-0.29, 0.02)	-0.19 (-0.35, -0.03)
Skoal	-0.16 (-0.41, 0.09)	-0.17 (-0.33, -0.01)	-0.20 (-0.36, -0.03)	-0.26 (-0.42, -0.10)
**Flavor**				
Tobacco	Ref.	Ref.	Ref.	Ref.
Wintergreen	0.66 ( 0.34, 1.04)	-0.47 (-0.63, -0.31)	-0.18 (-0.35, 0.00)	0.27 ( 0.08, 0.45)
Mint	1.10 ( 0.77, 1.42)	-0.42 (-0.59, -0.24)	-0.12 (-0.29, 0.05)	0.49 ( 0.31, 0.68)
Fruit	1.18 ( 0.86, 1.51)	-0.61 (-0.78, -0.44)	-0.33 (-0.51, -0.15)	0.47 ( 0.29, 0.65)
**Cut**				
Fine	Ref.	Ref.	Ref.	Ref.
Long	0.05 (-0.19, 0.28)	0.24 ( 0.07, 0.41)	0.14 (-0.03, 0.30)	-0.31 (-0.49, -0.14)
Wide	-0.02 (-0.25, 0.20)	0.25 ( 0.09, 0.42)	0.18 ( 0.02, 0.34)	-0.48 (-0.67, -0.29)
Pouch	-0.01 (-0.25, 0.22)	0.17 ( 0.00, 0.33)	0.11 (-0.04, 0.27)	0.04 (-0.13, 0.22)
**Price** (US$)				
3	Ref.	Ref.	Ref.	Ref.
5	0.18 (-0.04, 0.40)	0.08 (-0.07, 0.23)	0.41 ( 0.25, 0.57)	-0.06 (-0.20, 0.08)
8	0.35 ( 0.14, 0.55)	0.42 ( 0.26, 0.59)	0.71 ( 0.55, 0.87)	-0.23 (-0.40, -0.07)
**Position**				
Left	Ref.	Ref.	Ref.	Ref.
Right	-0.20 (-0.38, -0.02)	0.04 (-0.06, 0.15)	0.06 (-0.05, 0.17)	-0.04 (-0.15, 0.07)

Table reports coefficients from conditional logistic regression models in a discrete choice experiment. Coefficients indicate how much the attribute level in question (e.g. flavor: fruit) increased or decreased the log-odds of a displayed moist snuff product being chosen relative to the reference level (e.g. flavor: tobacco), adjusted for all other displayed moist snuff attributes. For each displayed pair of moist snuff products, participants were asked about which they were more curious, and which was more dangerous, would give a greater buzz, and would be easier to use. Positive values indicate characteristics (relative to reference) that independently contributed to greater curiosity or perceived danger, buzz, and ease of use, respectively. CI: confidence interval. Ref.: reference.

Among all responses, the probability of choosing ‘neither of these options’ rather than selecting one of the two composite products varied by product and the question being asked. In the e-cigarette experiment, participants indicated ‘neither’ most often when asked about which of the two products they were more curious (63%). ‘Neither’ was less often selected when asked about ease of use (39%), buzz (32%), and danger (26%). Similarly, in the smokeless tobacco experiment, ‘neither’ was indicated most often when asked about curiosity (81%), followed by ease-of-use (49%), buzz (48%), and danger (41%).

There was no statistically significant interaction (overall) by gender in the e-cigarette experiment (Supplementary file, Table A1). In contrast, having ever used an e-cigarette was associated with differences in all four perception outcomes (i.e. curiosity, danger, buzz, and ease-of-use; Supplementary file, Table A2). E-cigarette ever users held stronger perceptions about device types, viewing tank-type and pod-type devices with more curiosity relative to cigalike devices than did never users. Both e-cigarette ever and never users perceived flavored products with more curiosity and as easier to use compared to tobacco flavored products, but only never users believed that flavored products delivered less buzz than tobacco flavored e-cigarettes. Likewise, only never users were less curious about higher nicotine content e-cigarettes (Supplementary file, Table A2).

In the moist snuff experiment, the direction and magnitude of associations were similar by gender, but there was nominally statistically significant interaction for the outcomes curiosity and danger, as male participants indicated more curiosity about higher price products (Supplementary file, Table A3). Only 44 smokeless tobacco ever users completed the moist snuff experiment, limiting statistical power to detect differences in association by product use. Generally, brand perceptions were stronger among smokeless tobacco ever users. Only never users viewed flavored products as offering less buzz and as easier to use, whereas only ever users associated pouched products as easier to use (Supplementary file, Table A4).

## DISCUSSION

This study provides quantitative evidence that specific characteristics of non-cigarette tobacco products independently shape how youth perceive these products. As hypothesized, for both e-cigarettes and moist snuff smokeless tobacco, flavored products were viewed with more curiosity and as being less dangerous, less potent and easier to use compared to non-flavored products. Associations of flavors with greater curiosity and ease of use and less perceived danger held for all non-tobacco flavors, including mint and wintergreen. Thus, evidence from this crosssectional study population suggests that mint varieties should be included alongside fruit and dessert in flavor restrictions intended to reduce youth tobacco use^9^. Other product characteristics, such as e-cigarette device type, vapor amount and moist snuff price, also appear to shape product perceptions, which could inform tobacco control policy.

In January 2020, citing concern over growing youth e-cigarette use, the FDA announced a policy to prioritize enforcement of premarket authorization requirements for some types of e-cigarettes, but exempted mint and menthol flavors^18^. The present results suggest that youth perceive the properties of mint and wintergreen flavored tobacco similarly to fruit, dessert, and other flavors, which could undermine the effectiveness of the FDA policy. No such enforcement policy exists for conventional smokeless tobacco, but the present results demonstrate similar flavor association for moist snuff as observed for e-cigarettes. This finding is consistent with tobacco industry documents suggesting that flavored, lower priced, lower nicotine ‘starter products’ are used to target novice users before later ‘graduation’ to established use through a series of higher nicotine products^19,20^.

Use of flavored tobacco, including menthol, is more common among youth than adults^8^ and is the predominant way youth and young adults consumed tobacco across all tobacco products^8,9^. A review of qualitative studies reported that flavored tobacco (non-tobacco flavor) is viewed favorably by consumers, who associate flavors with less danger and often report that flavors contributed to their own tobacco experimentation and initiation^10^. Given the evidence that flavors contribute to youth use for all tobacco products^8,9^, current policies should consider not only restricting all non-tobacco flavors in cigarettes and e-cigarettes but in all forms of tobacco.

Pod-type e-cigarettes, such as market-leading brand JUUL, have become the most popular type of e-cigarette among US youth^21^. Independent of nicotine content and flavor, participants in the present study viewed pod devices with more curiosity and as easier to use than other device types but not necessarily as less dangerous. However, participants also associated low nicotine content e-cigarettes with less danger, more curiosity, and greater ease of use. Research suggests that youth may not recognize that pod-type e-cigarettes contain nicotine at high concentrations; a misperception potentially reinforced by the nicotine amount (‘5%’) printed on JUUL product packaging^22,23^.

Prior applications of discrete choice methods in tobacco control have focused on adults^5,6,24^ and/or recruited participants through online panels^6,7,24,25^. Previously reported findings include a preference for non-tobacco flavors both in e-cigarettes^6,25^ and waterpipe tobacco^5^, as well as identifying warning labels as a factor reducing product interest^5,25^. Consistent with the present study, Shang et al.^[Bibr cit0007]7^ reported that adolescents least prefer tobacco-flavored e-cigarettes and cigalike closed-system devices. The present study expands such work to moist snuff products, finds e-cigarette vapor cloud size and podtype devices as independent contributors to youth perceptions, and assesses the additional outcomes of perceived danger and ease of use. Furthermore, the present study shows no gender difference regarding e-cigarette perceptions, although beliefs associated with certain moist snuff product attributes were stronger among male participants, likely reflecting higher use and male-targeted marketing.

Not all perceived qualities observed in the present study aligned with actual product properties. Participants correctly associated higher nicotine content with stronger physical effects (‘buzz’). Other perceptions, such as flavored tobacco or small vapor cloud e-cigarettes being less dangerous, are not supported by scientific consensus^26^. This discordance between perceived and actual effects represents a possible area for corrective public messaging or for greater regulatory vigilance for potentially misleading marketing practices. Of note, and not unexpectedly, product ever users held stronger perceptions about device type (e-cigarettes) and brands (moist snuff) than did product naïve participants. In a crosssectional setting, it cannot be distinguished to what extent experience shaped perceptions or that existing attention to product attributes contributed to use initiation.

The designations curiosity, danger and ease-of-use are open to subjective interpretation. For example, danger could refer to either long-term or shortterm health effects. Meanwhile, ease of use could refer to concealability, access, adverse reactions, or social acceptance. While outcomes could be interpreted differently, they likely reflect multifaceted perceptions with plausible roles in decisionmaking. When considering risks and potential benefits of tobacco products, adolescents hold views that include multiple aspects of social and physical risks^27^. For e-cigarettes, specifically, adolescents cite multiple influences, both related to the product itself and their social context^13^. Adolescents’ smokeless tobacco use motivations likewise comprise multiple factors, including flavors, perceived nicotine strength, and loyalty to preferred brands^12^. The present study shows that several of these factors each independently contribute to multi-faceted perceptions.

Among these dimensions, curiosity and ease-ofuse are strong predictors of tobacco use behaviors. Tobacco product curiosity correlates with product susceptibility and use among youth^14,28^. Youth who perceived flavored smokeless tobacco and flavored e-cigarettes as easier to use than unflavored options were more likely to be susceptible to smokeless tobacco use^17^ and to initiate future e-cigarette use^16^, respectively. The present discrete choice findings demonstrate that multiple independent product-related factors are associated with constructs shown to predict future tobacco use.

### Strengths and limitations

A study limitation is that discrete choice experiments ask participants to make hypothetical choices that may not resemble the actual setting in which purchase or use decisions are made. Provided only limited information, study participants may have based some selections on word associations outside the context of e-cigarettes or moist snuff, for example, connecting the words ‘tobacco’ or ‘cigalike’ with dangers expressed in anti-smoking messages or considering ‘fine cut’ to indicate high quality rather than the coarseness of moist snuff tobacco. However, adolescents, especially those inexperienced with tobacco use, are likely to possess limited product information in real-word settings and may make the same cognitive associations when evaluating a tobacco product from words on a package, advertisement, or warning^29^. In this study, the presentation of images prior to initiating the textonly items may have helped mimic flavor imagery on packages or advertisements; however, it cannot be ruled out that the specific images or colors chosen had some influence on respondents’ choices.

A strength of discrete choice survey methods is that the contributions of multiple characteristics are considered in combination, corresponding better to real-world product choices. However, this strength relies on the unverifiable assumption that participants make rational trade-offs between product characteristics when evaluating options. To our knowledge, this study represents the first to apply discrete choice techniques to a school-based sample of youth for both smokeless tobacco and e-cigarettes, including pod-type devices. A school-based design may yield a more representative sample in terms of social-economic profile and tobacco use experiences than an online panel. However, as a limitation, data collected in rural regions of California may not generalize nationally, including to other rural geographical locations. As a non-random sample, the generalizability of this study population is also limited. Advantageously, a rural sample is likely to have greater familiarity with moist snuff products. However, the small total number of moist snuff users did not yield ideal power to examine interactions by use status. Notably, public messaging from health authorities in California emphasizing the potential harms of e-cigarettes and nicotine could have resulted in more concern about nicotine in this sample than would be observed elsewhere.

## CONCLUSIONS

Each e-cigarette and moist snuff characteristic assessed in this study of adolescents was independently associated with multiple product perceptions that presage youth tobacco use. For both e-cigarettes and moist snuff smokeless tobacco, all non-tobacco flavors were associated with more curiosity and perceived ease-of-use and lower perceived danger and physical effects. These findings suggest that all flavors, including mint, increase the youth appeal of both e-cigarettes and smokeless tobacco. Such youth perceptions should be considered in any decision to grant marketing authorization for a novel tobacco product. Given that all non-tobacco flavors were perceived favorably, both for e-cigarettes and moist snuff, this study provides evidence that would support restrictions of all non-tobacco flavors in all tobacco products in order to reduce the appeal of tobacco to youth.
