# Supplementary material for: Electronic cigarette and moist snuff product characteristics independently associated with youth tobacco product perceptions
Source: Tob Induc Dis. 2020 Aug 28;18:71. doi: 10.18332/tid/125513 (PMC7485438; doi:10.18332/tid/125513)
Supplement: Supplementary file 1 [file TID-18-71-S1.pdf]

APPENDIX: SUPPLEMENTAL FILES

Electronic Cigarette and Moist Snuff Product Characteristics Independently Associated with Youth Product Beliefs

Figure A.1. E-cigarette characteristics and levels displayed to discrete choice experiment participants

Figure A.2. Moist snuff smokeless tobacco characteristics and levels displayed to discrete choice experiment participants

Figure A.3. Example questionnaire item displayed to participants

Table A.1. Adolescents' beliefs associated with e-cigarette product characteristics: interaction by gender

Table A.2. Adolescents' beliefs associated with e-cigarette product characteristics: interaction by e-cigarette ever use

Table A.3. Adolescents' beliefs associated with moist snuff product characteristics: interaction by gender

Table A.4. Adolescents' beliefs associated with moist snuff product characteristics: interaction by smokeless tobacco ever use

Figure A.1. E-cigarette characteristics and levels displayed to discrete choice experiment participants

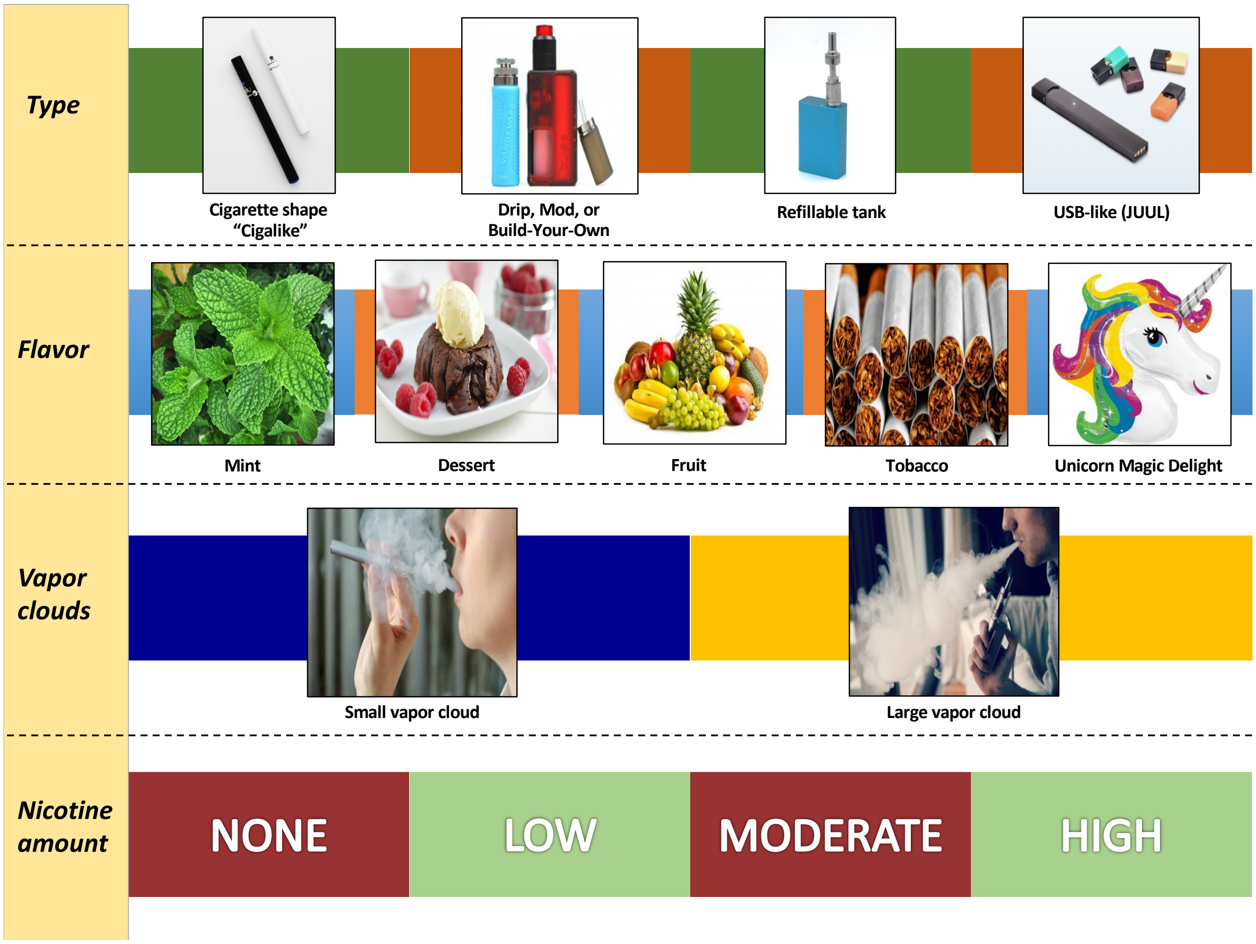

Legend: This image was displayed to survey participants in the e-cigarette discrete choice experiment to demonstrate the product characteristics and their possible levels that would appear in subsequent randomly-generated hypothetic e-cigarette composite products.

Figure A.2. Moist snuff smokeless tobacco characteristics and levels displayed to discrete choice experiment participants

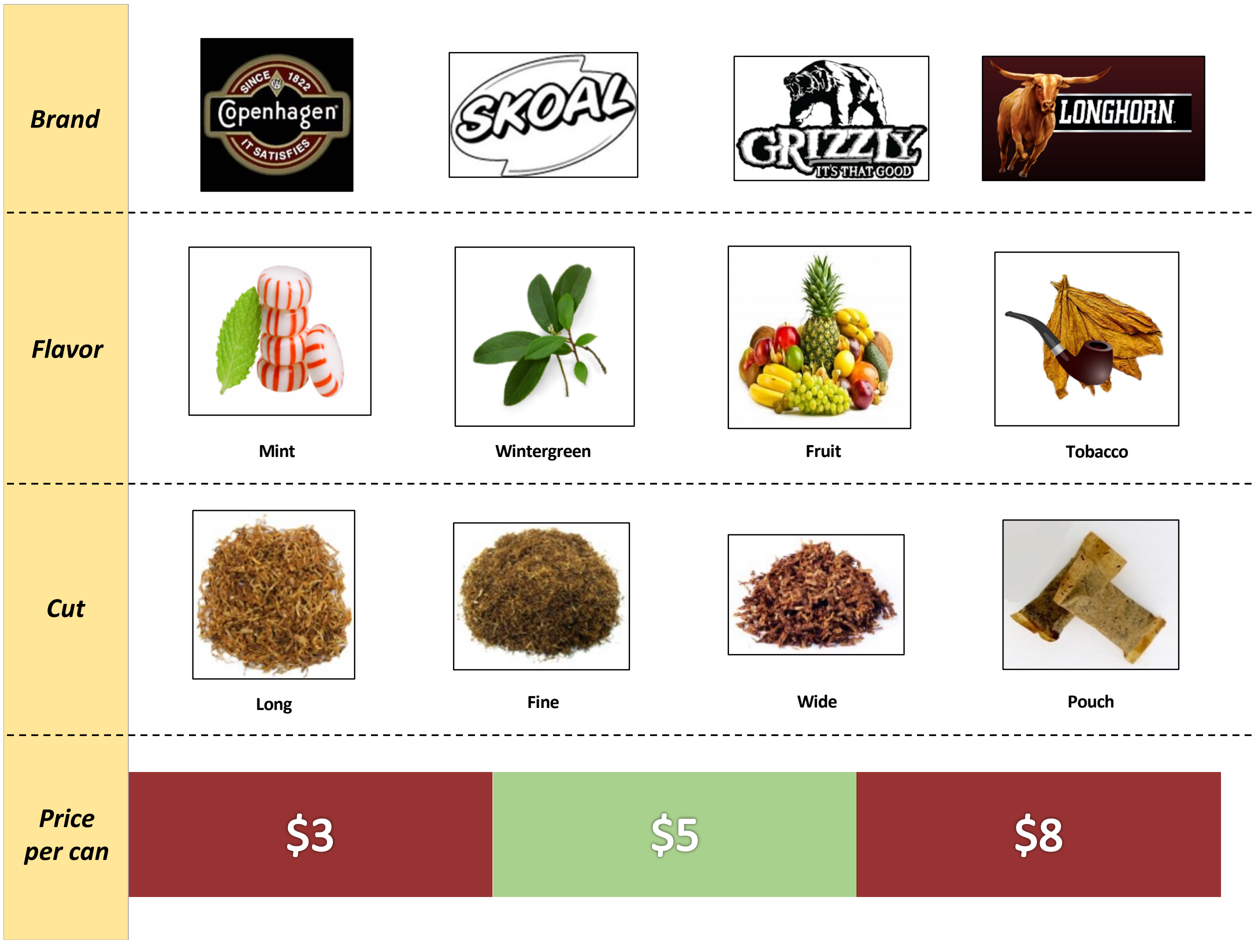

Legend: This image was displayed to survey participants in the moist snuff smokeless tobacco discrete choice experiment to demonstrate the product characteristics and their possible levels that would appear in subsequent randomly-generated hypothetic moist snuff composite products.

Figure A.3. Example questionnaire item displayed to participants

Of the following products, which one…?

	**Type:** tank or pen	**Type:** JUUL or other pod	
	**Flavor:** mint	**Flavor:** dessert	
	**Vapor Cloud:** large	**Vapor Cloud:** large	
	**Nicotine Amount:** high	**Nicotine Amount:** low	**Neither of these options**
**Are you more curious about?**	○	○	○
**Is more dangerous to health?**	○	○	○
**Would be easier to use?**	○	○	○
**Would give a bigger "buzz" or "head rush"?**	○	○	○

Legend: Discrete choice items were displayed to participants in the format above. Participants saw either e-cigarette items (above) or moist snuff smokeless tobacco items (not shown). Levels of product characteristics were randomly generated to construct each hypothetical product composite. Figure is simulated to enhance resolution: actual screen images differed slightly in presentation.

Table A.1. Adolescents' beliefs associated with e-cigarette product characteristics: interaction by gender

	**Curiosity** Coefficient (95% CI)		**Danger** Coefficient (95% CI)		**Buzz** Coefficient (95% CI)		**Ease of Use** Coefficient (95% CI)	
	P-for-interaction = 0.55		*P*-for-interaction = 0.49		*P*-for-interaction = 0.11		*P*-for-interaction = 0.79	
	**Female**	**Male**	**Female**	**Male**	**Female**	**Male**	**Female**	**Male**
**Device Type**								
Cigalike	reference	reference	reference	reference	reference	reference	reference	reference
Tank	0.35 (0.07, 0.62)	0.46 (0.16, 0.76)	-0.15 (-0.36, 0.05)	0.03 (-0.20, 0.26)	0.05 (-0.15, 0.25)	0.33 (0.06, 0.59)	0.21 (-0.03, 0.45)	0.24 (-0.01, 0.48)
Drip-mod	0.05 (-0.24, 0.33)	0.09 (-0.23, 0.41)	0.08 (-0.14, 0.30)	-0.03 (-0.24, 0.19)	0.06 (-0.16, 0.27)	0.27 (0.03, 0.51)	-0.21 (-0.46, 0.04)	-0.03 (-0.27, 0.21)
Pod	0.38 (0.13, 0.63)	0.39 (0.06, 0.71)	0.01 (-0.22, 0.24)	0.01 (-0.21, 0.23)	0.03 (-0.18, 0.24)	0.15 (-0.11, 0.39)	0.54 (0.32, 0.76)	0.62 (0.37, 0.88)
**Flavor**								
Tobacco	reference	reference	reference	reference	reference	reference	reference	reference
Dessert	1.10 (0.76, 1.44)	0.75 (0.40, 1.10)	-0.14 (-0.38, 0.11)	-0.34 (-0.59, -0.09)	-0.01 (-0.26, 0.23)	-0.15 (-0.40, 0.10)	0.52 (0.27, 0.77)	0.31 (0.02, 0.59)
Fruit	1.07 (0.73, 1.41)	1.00 (0.65, 1.36)	-0.20 (-0.45, 0.05)	-0.31 (-0.57, -0.04)	-0.09 (-0.33, 0.16)	-0.08 (-0.34, 0.17)	0.52 (0.28, 0.77)	0.46 (0.18, 0.73)
Mint	0.84 (0.48, 1.19)	0.67 (0.29, 1.04)	-0.27 (-0.49, -0.05)	-0.38 (-0.66, -0.10)	-0.08 (-0.32, 0.17)	-0.25 (-0.53, 0.02)	0.37 (0.11, 0.63)	0.34 (0.07, 0.62)
Unicorn	0.56 (0.21, 0.90)	0.70 (0.34, 1.06)	-0.24 (-0.46, -0.02)	-0.31 (-0.57, -0.04)	-0.10 (-0.33, 0.13)	-0.14 (-0.38, 0.10)	0.30 (0.05, 0.54)	0.25 (-0.03, 0.54)
**Vapor Cloud**								
Large	reference	reference	reference	reference	reference	reference	reference	reference
Small	-0.08 (-0.27, 0.11)	0.00 (-0.20, 0.20)	-0.32 (-0.49, -0.16)	-0.31 (-0.47, -0.14)	-0.35 (-0.51, -0.18)	-0.47 (-0.64, -0.29)	0.29 (0.14, 0.44)	0.24 (0.06, 0.41)
**Nicotine**								
None	reference	reference	reference	reference	reference	reference	reference	reference
Low	-0.10 (-0.35, 0.14)	-0.24 (-0.52, 0.03)	0.94 (0.68, 1.20)	0.77 (0.49, 1.06)	0.68 (0.44, 0.92)	0.51 (0.21, 0.80)	-0.09 (-0.31, 0.12)	-0.25 (-0.48, -0.02)
Moderate	-0.30 (-0.57, -0.03)	-0.32 (-0.62, -0.03)	1.65 (1.37, 1.93)	1.57 (1.27, 1.86)	1.31 (1.04, 1.57)	1.05 (0.76, 1.35)	-0.34 (-0.57, -0.10)	-0.49 (-0.76, -0.23)
High	-0.41 (-0.69, -0.14)	-0.62 (-0.98, -0.26)	2.24 (1.91, 2.58)	2.13 (1.79, 2.47)	1.86 (1.56, 2.15)	1.70 (1.36, 2.04)	-0.53 (-0.78, -0.28)	-0.54 (-0.81, -0.27)
**Position**								
Left	reference	reference	reference	reference	reference	reference	reference	reference
Right	-0.04 (-0.21, 0.12)	-0.08 (-0.28, 0.12)	0.06 (-0.06, 0.19)	-0.02 (-0.16, 0.12)	0.11 (-0.01, 0.24)	-0.06 (-0.20, 0.08)	-0.03 (-0.15, 0.09)	0.15 (0.00, 0.30)

Table reports coefficients from conditional logistic regression models in a discrete choice experiment. Interaction terms were added to assess differences by gender. Coefficients indicate how much the attribute level in question (e.g., flavor: fruit) increased or decreased the log-odds of a displayed e-cigarette product being chosen relative to the reference level (e.g., flavor: tobacco), adjusted for all other displayed e-cigarette attributes. For each displayed pair of e-cigarettes, participants were asked about which they were more curious, and which was more dangerous, would give a greater buzz, and would be easier to use. Positive values indicate characteristics (relative to reference) that independently contributed to greater curiosity or perceived danger, buzz, and ease of use, respectively.
Abbreviation: CI = Confidence interval

Table A.2. Adolescents' beliefs associated with e-cigarette product characteristics: interaction by e-cigarette ever use

	**Curiosity** Coefficient (95% CI)		**Danger** Coefficient (95% CI)		**Buzz** Coefficient (95% CI)		**Ease of Use** Coefficient (95% CI)	
	P-for-interaction < 0.0001		*P*-for-interaction = 0.009		*P*-for-interaction < 0.0001		*P*-for-interaction < 0.0001	
	**Never Used**	**Ever Used**	**Never Used**	**Ever Used**	**Never Used**	**Ever Used**	**Never Used**	**Ever Used**
**Device Type**								
Cigalike	reference	reference	reference	reference	reference	reference	reference	reference
Tank	-0.02 (-0.30, 0.26)	0.79 (0.50, 1.09)	-0.10 (-0.30, 0.11)	-0.04 (-0.27, 0.20)	0.07 (-0.14, 0.27)	0.30 (0.03, 0.56)	0.08 (-0.15, 0.31)	0.45 (0.18, 0.73)
Drip-mod	-0.22 (-0.50, 0.06)	0.30 (-0.02, 0.61)	-0.03 (-0.22, 0.17)	0.16 (-0.09, 0.42)	0.12 (-0.08, 0.32)	0.22 (-0.04, 0.48)	-0.26 (-0.48, -0.04)	0.02 (-0.26, 0.30)
Pod	0.22 (-0.06, 0.49)	0.54 (0.25, 0.83)	0.01 (-0.19, 0.21)	0.00 (-0.26, 0.27)	0.02 (-0.19, 0.23)	0.14 (-0.11, 0.39)	0.46 (0.25, 0.68)	0.73 (0.47, 0.99)
**Flavor**								
Tobacco	reference	reference	reference	reference	reference	reference	reference	reference
Dessert	0.46 (0.10, 0.82)	1.38 (1.02, 1.74)	-0.31 (-0.54, -0.08)	-0.13 (-0.41, 0.14)	-0.26 (-0.49, -0.04)	0.18 (-0.09, 0.45)	0.27 (0.03, 0.52)	0.62 (0.33, 0.90)
Fruit	0.81 (0.46, 1.16)	1.34 (0.98, 1.71)	-0.38 (-0.61, -0.16)	-0.03 (-0.34, 0.28)	-0.25 (-0.47, -0.03)	0.12 (-0.16, 0.40)	0.40 (0.16, 0.63)	0.64 (0.35, 0.94)
Mint	0.49 (0.11, 0.87)	1.13 (0.77, 1.49)	-0.40 (-0.62, -0.18)	-0.17 (-0.46, 0.12)	-0.38 (-0.62, -0.15)	0.23 (-0.05, 0.50)	0.22 (-0.02, 0.47)	0.59 (0.30, 0.89)
Unicorn	0.36 (0.00, 0.71)	0.96 (0.59, 1.33)	-0.40 (-0.61, -0.19)	-0.05 (-0.34, 0.25)	-0.34 (-0.55, -0.14)	0.22 (-0.06, 0.51)	0.03 (-0.21, 0.26)	0.67 (0.37, 0.98)
**Vapor Cloud**								
Large	reference	reference	reference	reference	reference	reference	reference	reference
Small	-0.08 (-0.28, 0.13)	0.01 (-0.19, 0.21)	-0.33 (-0.48, -0.18)	-0.29 (-0.48, -0.10)	-0.44 (-0.60, -0.29)	-0.33 (-0.52, -0.14)	0.24 (0.09, 0.40)	0.33 (0.15, 0.51)
**Nicotine**								
None	reference	reference	reference	reference	reference	reference	reference	reference
Low	-0.39 (-0.66, -0.11)	0.01 (-0.24, 0.26)	0.86 (0.61, 1.10)	0.85 (0.55, 1.15)	0.58 (0.34, 0.83)	0.59 (0.29, 0.88)	-0.21 (-0.42, 0.00)	-0.11 (-0.36, 0.13)
Moderate	-0.67 (-0.98, -0.37)	-0.07 (-0.34, 0.21)	1.54 (1.29, 1.79)	1.68 (1.35, 2.01)	1.15 (0.89, 1.41)	1.22 (0.91, 1.52)	-0.48 (-0.71, -0.24)	-0.36 (-0.62, -0.10)
High	-0.91 (-1.24, -0.57)	-0.24 (-0.55, 0.07)	2.10 (1.82, 2.39)	2.24 (1.85, 2.64)	1.73 (1.45, 2.01)	1.82 (1.44, 2.19)	-0.63 (-0.87, -0.39)	-0.44 (-0.74, -0.15)
**Position**								
Left	reference	reference	reference	reference	reference	reference	reference	reference
Right	-0.01 (-0.21, 0.19)	-0.06 (-0.23, 0.11)	0.03 (-0.09, 0.15)	0.05 (-0.11, 0.20)	0.04 (-0.07, 0.16)	0.04 (-0.12, 0.20)	-0.03 (-0.16, 0.09)	0.17 (0.02, 0.31)

Table reports coefficients from conditional logistic regression models in a discrete choice experiment. Interaction terms were added to assess differences by having ever used an e-cigarette. Coefficients indicate how much the attribute level in question (e.g., flavor: fruit) increased or decreased the log-odds of a displayed e-cigarette product being chosen relative to the reference level (e.g., flavor: tobacco), adjusted for all other displayed e-cigarette attributes. For each displayed pair of e-cigarettes, participants were asked about which they were more curious, and which was more dangerous, would give a greater buzz, and would be easier to use. Positive values indicate characteristics (relative to reference) that independently contributed to greater curiosity or perceived danger, buzz, and ease of use, respectively.
Abbreviation: CI = Confidence interval

Table A.3. Adolescents' beliefs associated with moist snuff product characteristics: interaction by gender

	Curiosity<br>Coefficient (95% CI)		Danger<br>Coefficient (95% CI)		Buzz<br>Coefficient (95% CI)		Ease of Use<br>Coefficient (95% CI)	
	P-for-interaction = 0.009		*P*-for-interaction = 0.003		*P*-for-interaction = 0.88		*P*-for-interaction = 0.85	
	**Female**	**Male**	**Female**	**Male**	**Female**	**Male**	**Female**	**Male**
**Brand**								
Copenhagen	reference	reference	reference	reference	reference	reference	reference	reference
Grizzly	-0.05 (-0.37, 0.27)	0.11 (-0.26, 0.48)	-0.18 (-0.41, 0.04)	-0.40 (-0.67, -0.14)	-0.05 (-0.26, 0.15)	-0.20 (-0.45, 0.05)	-0.23 (-0.45, -0.01)	-0.39 (-0.63, -0.14)
Longhorn	-0.03 (-0.37, 0.31)	-0.20 (-0.58, 0.18)	-0.27 (-0.48, -0.06)	-0.18 (-0.41, 0.04)	-0.07 (-0.28, 0.14)	-0.22 (-0.44, 0.01)	-0.18 (-0.39, 0.04)	-0.19 (-0.42, 0.04)
Skoal	-0.09 (-0.39, 0.21)	-0.26 (-0.64, 0.12)	-0.20 (-0.40, 0.01)	-0.18 (-0.42, 0.07)	-0.13 (-0.34, 0.09)	-0.30 (-0.54, -0.06)	-0.25 (-0.46, -0.04)	-0.27 (-0.51, -0.03)
**Flavor**								
Tobacco	reference	reference	reference	reference	reference	reference	reference	reference
Wintergreen	0.84 (0.37, 1.32)	0.63 (0.18, 1.07)	-0.49 (-0.70, -0.28)	-0.45 (-0.69, -0.21)	-0.16 (-0.39, 0.07)	-0.19 (-0.44, 0.06)	0.19 (-0.07, 0.44)	0.37 (0.12, 0.62)
Mint	1.25 (0.82, 1.67)	1.05 (0.63, 1.48)	-0.61 (-0.85, -0.37)	-0.15 (-0.39, 0.09)	-0.15 (-0.37, 0.07)	-0.05 (-0.29, 0.18)	0.45 (0.18, 0.72)	0.53 (0.30, 0.77)
Fruit	1.61 (1.18, 2.03)	0.79 (0.35, 1.23)	-0.66 (-0.87, -0.45)	-0.51 (-0.78, -0.25)	-0.25 (-0.49, -0.01)	-0.39 (-0.64, -0.14)	0.48 (0.23, 0.73)	0.47 (0.23, 0.70)
**Cut**								
Fine	reference	reference	reference	reference	reference	reference	reference	reference
Long	0.15 (-0.13, 0.43)	-0.08 (-0.46, 0.31)	0.37 (0.15, 0.59)	0.10 (-0.15, 0.35)	0.12 (-0.10, 0.34)	0.17 (-0.08, 0.43)	-0.37 (-0.60, -0.13)	-0.25 (-0.52, 0.01)
Wide	-0.03 (-0.32, 0.25)	-0.03 (-0.39, 0.32)	0.38 (0.15, 0.61)	0.10 (-0.14, 0.34)	0.22 (0.01, 0.43)	0.13 (-0.12, 0.38)	-0.57 (-0.83, -0.32)	-0.39 (-0.64, -0.14)
Pouch	-0.19 (-0.51, 0.14)	0.14 (-0.20, 0.47)	0.23 (0.01, 0.44)	0.11 (-0.14, 0.36)	0.04 (-0.17, 0.24)	0.21 (-0.02, 0.45)	-0.02 (-0.25, 0.21)	0.12 (-0.13, 0.38)
**Price**								
$3	reference	reference	reference	reference	reference	reference	reference	reference
$5	0.02 (-0.23, 0.27)	0.36 (-0.01, 0.74)	0.15 (-0.05, 0.36)	0.01 (-0.20, 0.22)	0.37 (0.15, 0.59)	0.44 (0.21, 0.68)	-0.04 (-0.24, 0.16)	-0.08 (-0.29, 0.13)
$8	0.17 (-0.11, 0.46)	0.53 (0.24, 0.82)	0.55 (0.32, 0.78)	0.28 (0.04, 0.51)	0.68 (0.46, 0.90)	0.71 (0.47, 0.94)	-0.19 (-0.42, 0.04)	-0.26 (-0.49, -0.04)
**Position**								
Left	reference	reference	reference	reference	reference	reference	reference	reference
Right	-0.38 (-0.62, -0.13)	-0.02 (-0.28, 0.25)	0.08 (-0.06, 0.22)	0.01 (-0.14, 0.16)	0.05 (-0.10, 0.20)	0.05 (-0.12, 0.22)	-0.07 (-0.21, 0.07)	-0.01 (-0.18, 0.16)

Table reports coefficients from conditional logistic regression models in a discrete choice experiment. Interaction terms were added to assess differences by gender. Coefficients indicate how much the attribute level in question (e.g., flavor: fruit) increased or decreased the log-odds of a displayed moist snuff product being chosen relative to the reference level (e.g., flavor: tobacco), adjusted for all other displayed product attributes. For each displayed pair of moist snuff products, participants were asked about which they were more curious, and which was more dangerous, would give a greater buzz, and would be easier to use. Positive values indicate characteristics (relative to reference) that independently contributed to greater curiosity or perceived danger, buzz, and ease of use, respectively.
Abbreviation: CI = Confidence interval

Table A.4. Adolescents' beliefs associated with moist snuff product characteristics: interaction by smokeless tobacco ever use

	Curiosity<br>Coefficient (95% CI)		Danger<br>Coefficient (95% CI)		Buzz<br>Coefficient (95% CI)		Ease of Use<br>Coefficient (95% CI)	
	P-for-interaction < 0.0001		*P*-for-interaction = 0.24		*P*-for-interaction = 0.68		*P*-for-interaction = 0.11	
	**Never Used**	**Ever Used**	**Never Used**	**Ever Used**	**Never Used**	**Ever Used**	**Never Used**	**Ever Used**
**Brand**								
Copenhagen	reference	reference	reference	reference	reference	reference	reference	reference
Grizzly	0.07 (-0.21, 0.34)	-0.36 (-1.02, 0.30)	-0.27 (-0.45, -0.09)	-0.52 (-1.15, 0.12)	-0.10 (-0.27, 0.06)	-0.33 (-0.91, 0.24)	-0.28 (-0.46, -0.11)	-0.53 (-1.05, -0.01)
Longhorn	-0.09 (-0.37, 0.19)	-0.47 (-1.15, 0.20)	-0.18 (-0.34, -0.02)	-0.62 (-1.15, -0.08)	-0.11 (-0.27, 0.05)	-0.50 (-1.11, 0.12)	-0.16 (-0.33, 0.01)	-0.47 (-0.95, 0.00)
Skoal	-0.08 (-0.34, 0.18)	-0.80 (-1.55, -0.06)	-0.14 (-0.31, 0.03)	-0.58 (-1.14, -0.03)	-0.16 (-0.33, 0.01)	-0.62 (-1.22, -0.02)	-0.21 (-0.37, -0.05)	-0.89 (-1.59, -0.20)
**Flavor**								
Tobacco	reference	reference	reference	reference	reference	reference	reference	reference
Wintergreen	0.63 (0.27, 1.00)	1.11 (0.24, 1.97)	-0.48 (-0.65, -0.32)	-0.28 (-0.77, 0.26)	-0.20 (-0.38, -0.02)	0.16 (-0.42, 0.75)	0.29 (0.10, 0.48)	-0.01 (-0.61, 0.60)
Mint	1.06 (0.73, 1.40)	1.41 (0.59, 2.24)	-0.41 (-0.59, -0.22)	-0.53 (-1.12, 0.06)	-0.12 (-0.29, 0.06)	-0.10 (-0.51, 0.31)	0.51 (0.32, 0.70)	0.30 (-0.30, 0.91)
Fruit	1.19 (0.85, 1.53)	1.29 (0.43, 2.16)	-0.63 (-0.81, -0.46)	-0.27 (-0.86, 0.33)	-0.36 (-0.54, -0.17)	0.03 (-0.52, 0.58)	0.53 (0.34, 0.72)	-0.13 (-0.57, 0.32)
**Cut**								
Fine	reference	reference	reference	reference	reference	reference	reference	reference
Long	-0.03 (-0.28, 0.22)	0.62 (-0.10, 1.33)	0.25 (0.07, 0.43)	0.10 (-0.43, 0.63)	0.15 (-0.02, 0.32)	0.01 (-0.70, 0.73)	-0.35 (-0.53, -0.16)	0.09 (-0.64, 0.82)
Wide	-0.06 (-0.31, 0.18)	0.26 (-0.41, 0.92)	0.28 (0.10, 0.45)	0.03 (-0.45, 0.51)	0.19 (0.01, 0.36)	0.13 (-0.38, 0.65)	-0.52 (-0.71, -0.32)	-0.02 (-0.64, 0.61)
Pouch	-0.14 (-0.39, 0.12)	0.70 (0.05, 1.34)	0.20 (0.02, 0.37)	-0.16 (-0.61, 0.30)	0.11 (-0.06, 0.27)	0.18 (-0.31, 0.67)	-0.02 (-0.20, 0.15)	0.74 (0.12, 1.36)
**Price**								
$3	reference	reference	reference	reference	reference	reference	reference	reference
$5	0.12 (-0.12, 0.36)	0.53 (-0.09, 1.15)	0.09 (-0.07, 0.24)	-0.03 (-0.63, 0.56)	0.42 (0.25, 0.58)	0.43 (-0.14, 0.99)	-0.06 (-0.21, 0.09)	0.00 (-0.50, 0.49)
$8	0.29 (0.06, 0.52)	0.81 (0.33, 1.28)	0.41 (0.24, 0.59)	0.54 (-0.03, 1.11)	0.70 (0.55, 0.87)	0.82 (0.33, 1.30)	-0.25 (-0.42, -0.08)	-0.06 (-0.58, 0.47)
**Position**								
Left	reference	reference	reference	reference	reference	reference	reference	reference
Right	-0.28 (-0.47, -0.09)	0.24 (-0.36, 0.83)	0.04 (-0.06, 0.15)	0.06 (-0.30, 0.43)	0.08 (-0.03, 0.20)	-0.14 (-0.57, 0.29)	-0.04 (-0.15, 0.07)	-0.01 (-0.50, 0.47)

Table reports coefficients from conditional logistic regression models in a discrete choice experiment. Interaction terms were added to assess differences by having ever used conventional smokeless tobacco. Coefficients indicate how much the attribute level in question (e.g., flavor: fruit) increased or decreased the log-odds of a displayed moist snuff product being chosen relative to the reference level (e.g., flavor: tobacco), adjusted for all other displayed product attributes. For each displayed pair of moist snuff products, participants were asked about which they were more curious, and which was more dangerous, would give a greater buzz, and would be easier to use. Positive values indicate characteristics (relative to reference) that independently contributed to greater curiosity or perceived danger, buzz, and ease of use, respectively.
Abbreviation: CI = Confidence interval

© 2020 Chaffee B.W. et al.
